# Exosomes from Kartogenin-Pretreated Infrapatellar Fat Pad Mesenchymal Stem Cells Enhance Chondrocyte Anabolism and Articular Cartilage Regeneration

**DOI:** 10.1155/2021/6624874

**Published:** 2021-03-09

**Authors:** Jiahua Shao, Jun Zhu, Yi Chen, Qiwei Fu, Lexiang Li, Zheru Ding, Jun Wu, Yaguang Han, Haobo Li, Qirong Qian, Yiqin Zhou

**Affiliations:** Department of Orthopedics, Shanghai Changzheng Hospital, Naval Medical University, Shanghai 200003, China

## Abstract

**Objective:**

To evaluate the effect of Kartogenin-pretreated exosomes derived from infrapatellar fat pad mesenchymal stem cells on chondrocyte in vitro and articular cartilage regeneration in vivo.

**Methods:**

Infrapatellar fat pad mesenchymal stem cells (IPFP-MSCs) were isolated from rabbits to harvest exosomes. After identification of mesenchymal stem cells and exosomes, rabbit chondrocytes were divided into three groups for further treatment: the EXO group (chondrocytes treated with exosomes isolated from infrapatellar fat pad mesenchymal stem cells), KGN-EXO group (chondrocytes treated with exosomes isolated from infrapatellar fat pad mesenchymal stem cells pretreated with KGN), and control group. After processing and proliferation, phenotypic changes of chondrocytes were measured. In the in vivo study, 4 groups of rabbits with articular cartilage injury were treated with KGN-EXO, EXO, IPFP-MSCs, and control. Macroscopic evaluation and histological evaluation were made to figure out the different effects of the 4 groups on cartilage regeneration in vivo.

**Results:**

The proliferation rate of chondrocytes in the EXO or KGN-EXO group was significantly higher than that in the control group (*P* < 0.05). The qRT-PCR results showed that the expression of Sox-9, Aggrecan, and Col II was the highest in the KGN-EXO group compared with the EXO group and the control group (*P* < 0.05). The results of Western blot were consistent with the results of qRT-PCR. In vivo, the cartilage defects in the KGN-EXO group showed better gross appearance and improved histological score than those in IPFP-MSC groups, EXO groups, and control groups (*P* < 0.05). At 12 weeks, the defect site in the KGN-EXO group was almost completely repaired with a flat and smooth surface, while a large amount of hyaline cartilage-like structures and no obvious cracks were observed.

**Conclusion:**

Our study demonstrates that the exosomes isolated from infrapatellar fat pad mesenchymal stem cells pretreated with KGN have potent ability to induce chondrogenic differentiation of stem cells, effectively promoting the proliferation and the expression of chondrogenic proteins and genes of chondrocytes. The KGN-EXO can also promote the repair of articular cartilage defects more effectively, which can be used as a potential therapeutic method in the future.

## 1. Introduction

Osteoarthritis (OA) is one of the most common diseases encountered in the field of orthopedics. OA is a chronic degenerative joint disease that commonly causes pain and limited mobility. Furthermore, the treatment costs associated with OA are high [1]. OA is characterized by the loss of extracellular matrix and the destruction of articular cartilage [2, 3]. There are many risk factors associated with OA, including genetic factors, female gender, a history of trauma, age, and obesity [4]. Currently, there are approximately 237 million people worldwide suffering from OA [5, 6]. The main pathologic change of OA is articular cartilage lesion. Promoting articular cartilage repair or regeneration is the key to prevent OA progress. However, it is a worldwide challenge to promote articular cartilage regeneration or repair through current clinical methods, such as medications, physical therapy, arthroscopy, microfracture, or cartilage transplantation [7, 8].

For the past few years, there have been significant advancements in the field of tissue engineering and regenerative medicine. These forms of biological treatment could represent a novel and promising way to help regenerate articular cartilage. Several studies have shown that lesions in articular cartilage could be regenerated effectively by biological intervention *in vitro* or *in vivo*, including mesenchymal stem cells (MSCs), biological growth factors, or other tissue engineering methods [9, 10]. In particular, MSCs have been proved to be particularly promising for the repair of lesions in articular cartilage lesion, as demonstrated by a combination of both basic and clinical research. Although some of these previous studies have reported exciting results, there are still significant problems remaining if we are to apply MSCs to the treatment of patients in the early stage of OA, including inconsistent data, the poor quality of autologous MSCs, ethical issues relating to xenogenous MSCs, and the risk of tumorigenicity or infection [11]. Collectively, these issues create a significant limitation to the widespread and consistent application of MSCs in the clinical treatment of OA.

Recently, Murphy et al. suggested that the mechanism underlying the use of MSCs to repair damaged tissues is not mainly related to their capacity to promote the differentiation of MSCs but rather via the paracrine pathways associated with these cells [12]. Extracellular vesicles secreted by the paracrine pathway release a variety of cytokines by binding to target cells. These cytokines subsequently regulate tissue regeneration [13, 14]. Existing research indicates that exosomes are the most important form of these extracellular vesicles. Exosomes are 40 to 120 nm in diameter and contain a large number of proteins, nucleic acids, lipids, and other components secreted by cells. There are extensive differences in the components of exosomes secreted by different cell types and even by the same cell type under different conditions [15, 16]. Some studies have found that exosomes extracted from cells have a more targeted effect when pretreated by specific methodology [17]. Kartogenin (KGN) is a small molecular compound that was identified in over 22,000 heterocyclic drug molecules by Johnson et al. [18]. In their research, KGN could effectively promote the differentiation of MSCs specifically into chondrocytes. So, it is quite interesting to explore the paracrine changes of MSCs pretreated by KGN. Thus, in this study, MSCs derived from the infrapatellar fat pad of rabbits were pretreated with KGN and their exosomes were extracted for comparison with MSCs-exosomes without treating. The main aim is to investigate the role of two kinds of exosomes in the promotion of cartilage repair *in vitro* and *in vivo*.

## 2. Methods and Materials

### 2.1. Ethics Statement

All animal procedures of this study were conducted with the approval of the Ethics Committee of Second Military Medical University and in compliance with the Institutional Animal Care and Use Committee (IACUC), following international guidelines for animal treatment.

### 2.2. Preparation of MSCs and Chondrocytes

MSCs and chondrocytes were obtained from New Zealand white rabbits (aged less than 6 months and weighing between 2 kg and 2.5 kg). From each rabbit, we removed the infrapatellar fat pad and knee cartilage block. Next, we removed blood vessels and connective tissue and used enzymatic methods to isolate infrapatellar fat pad mesenchymal stem cells (IPFP-MSCs) and chondrocytes, as described previously [19]. The MSCs were then subjected to flow cytometry to detect a variety of surface antibodies (CD34, CD45, CD73, CD90, and CD105); IgG1-PE was used as a negative control to exclude potential interference from fluorescein. In addition, MSCs were cultured in three stages of differentiation: osteogenic, adipogenic, and chondrogenic stages, in order to identify their relative potential to differentiate in different directions.

### 2.3. Western Blotting

Western blotting was carried out as described previously [20]. In brief, PMSF-RIPA lysis buffer was added to the cells and the resultant lysate was centrifuged at 12,000 rpm for 5 mins to permit collection of the supernatant. The concentration of each protein was measured by the BCA method and the concentrations were adjusted to separation by SDS-PAGE. For each sample, we loaded 15 *μ*l (50 *μ*g) per well. Following separation, proteins were transferred to the nitrocellulose membrane. Membranes were then incubated with primary antibodies (anti-CD9, CBL162, Sigma-Aldrich; anti-TSG101, SAB2702167, Sigma-Aldrich; anti-GAPDH, G8795, Sigma-Aldrich; anti-PPAR*γ*, MAB3872, Sigma-Aldrich; anti-Col2, CP18, Sigma-Aldrich; anti-Runx2, AV36678, Sigma-Aldrich; anti-Sox9, AV37986, Sigma-Aldrich; and anti-Aggrecan, MABT83, Sigma-Aldrich) at 1 : 1,000 and secondary antibodies at 1 : 5,000 (goat anti-rabbit (ab6721, Abcam) or goat anti-mouse (ab97023, Abcam) horseradish peroxidase- (HRP-) conjugated secondary antibody), and positive binding was visualized using a ChemiDoc™ XRS imaging system (Bio-Rad, Beijing, China). The immunoreactive bands were analyzed with ImageJ software (National Institutes of Health, Bethesda, MD, USA).

### 2.4. Real-Time Reverse Transcription-Polymerase Chain Reaction (qRT-PCR)

Total RNA was extracted from cells using TRIzol (Invitrogen, Shanghai, China), in accordance with the manufacturer's instructions. RNA was then reverse transcribed using qScript cDNA SuperMix reagent (Quanta BioSciences, Beijing, China), and relative gene expression was determined by qRT-PCR and the 2^−ΔΔCT^ method. The primer sequences were as follows: *SOX9* (5′-AGCAAGAACAAGCCCCACGTC-3′, 5′CCTGCCCATTCTTCACCGACT-3′); *ACAN* (5′-CATCTGGAGTTCTTTTTGGGAG-3′, 5′-CAGGTCAGGGATTCTGTGTGTC-3′); *COL2A1* (5′-GAAGACACCAAGGACTGCCTG-3′, 5′-GCACCCTTTTCGCCTTTGTCA-3′); *PPARγ* (5′-TGCAGGAGCAGAGCAAAGAAG-3′, 5′-GAGGCCAGCATGGTGTAGATG-3′); and *Runx-2* (5′-TGATGACACTGCCACCTGTG-3′, 5′-ACTCTGGCTTTGGGAAGAGC-3′). Each experiment was repeated in triplicate.

### 2.5. Extraction, Identification, and Measurement of Exosomes

IPFP-MSCs from passage 3 were selected and cultured in two groups. One group was cultured normally, while the other group was cultured with 5 *μ*l of 10 mmol/L KGN in 5 ml of medium as a pretreatment. After 72 hours of culture, the supernatant was extracted and stored at −80°C. Exosomes (MSC-EXOs) were then extracted using multiple rounds of centrifugation. First cells were centrifuged at 300 g for 10 minutes. The supernatant was then collected and centrifuged at 2,000 g for 10 minutes. Again, the supernatant was collected and centrifuged at 10,000 g for 30 minutes. The supernatant was collected and then recentrifuged at 100,000 g for 70 minutes. The supernatant was then discarded, and the pellet was resuspended with phosphate-buffered saline (PBS); this was then centrifuged at 100,000 g for 70 minutes. Finally, the supernatant was discarded, and the precipitate was resuspended in 200 *μ*l of PBS and stored at −80°C. For analysis, 10 *μ*l of exosome solution was then added to a copper mesh and examined by electron microscopy. We then used Western blotting to determine the expression of CD9 and TSG101 on the surface of the extracted exosomes. The size distribution of the extracted exosomes was then determined using a NanoSight NS300 system (Malvern Panalytical, Malvern, UK).

### 2.6. The Effect of Exosomes on Cell Proliferation

Next, we selected chondrocytes showing good rates of growth from passage 3 and divided these into three groups. Chondrocytes in the EXO group were treated with 1 × 10^8^ IPFP-MSCs. Chondrocytes in the KGN-EXO group were treated with 1 × 10^8^ IPFP-MSC exosomes and KGN as a pretreatment. Finally, chondrocytes in the control group were treated with PBS as a blank control. CCK-8 reagent was subsequently used to detect cell proliferation in each group for 7 consecutive days.

### 2.7. The Effect of Exosomes on the Phenotype of Chondrocytes

Chondrocytes from passage 3 were selected and divided into the same three groups as described above and cultured at a 37°C temperature for 14 days. Chondrocytes were then collected from each of the three groups. We then determined the relative expression levels of Sox-9, Aggrecan, Col-II, PPAR*γ*, and Runx-2, by Western blotting and qRT-PCR.

### 2.8. The Establishment of the Rabbit Articular Cartilage Injury Model

Forty-eight healthy New Zealand white rabbits (aged 5–6 months and weighing between 2 and 2.5 kg) were used for the in vivo study. All animals were treated with care at all times, and all experimental procedures were approved by the ethics committee and carried out in strict accordance with the ethical rules governing animal experimentation. For consistency, the right knee was selected as the experimental surgical site in order to create a rabbit model of knee cartilage injury. The 48 rabbits were divided into 4 groups at random. The control group received an intra-articular injection of 0.5 ml PBS, the IPFP-MSC group received an intra-articular injection of cell suspension containing 1 × 10^7^ IPFP-MSCs, the EXO group received an intra-articular injection of suspension containing 1 × 10^10^ Exos, and the KGN-EXO group received an intra-articular injection of suspension containing 1 × 10^10^ KGN-Exos. The rabbit model of knee cartilage injury was created as follows. First, all rabbits were anesthetized by slowly injecting sodium pentobarbital into the ear vein. Then, penicillin was slowly administered to prevent infection. Rabbits were then placed in a supine position and a medial parapatellar approach was used to open the joint capsule. The patella was then pulled laterally to expose the femoral trochlea. A cartilage defect (4 mm in diameter and 1.5 mm in depth) was then drilled into the center of the femoral trochlea using a sterile electric drill. Thereafter, penicillin sodium was injected daily into the gluteus maximus to prevent infection for the first 3 days after surgery. During this time, rabbits were not restricted and were allowed to be active. Six experimental animals from each group were sacrificed at 4 and 12 weeks after surgery for analysis.

### 2.9. Macroscopic Evaluation

Rabbits were sacrificed by an intravenous injection of sodium pentobarbital. The surgical site was then exposed and harvested. The cartilage defect sites were then photographed and evaluated in a blinded manner in accordance with the International Cartilage Repair Society (ICRS) scoring system (Table 1). Scoring was carried out independently by three investigators.

### 2.10. Histological Evaluation

Specimens were fixed in 4% paraformaldehyde for 36 hours and then decalcified with 20% EDTA solution at room temperature for 4–6 weeks. Samples were then measured by needle punching every 2 weeks until the needle could be easily inserted into the bone tissue, thus indicating that decalcification was complete. The samples were then dehydrated with a gradient series of alcohols, embedded in paraffin, and sectioned to create histological sections that were 4 *μ*m thick. Sections were then stained with HE and Safranin O/Fast Green. In order to achieve consistent and objective results, the sections were then evaluated using the modified O'Driscoll histological score (Table 2) [21].

## 3. Results

### 3.1. Characterization of IPFP-MSCs

**Figure 1:**
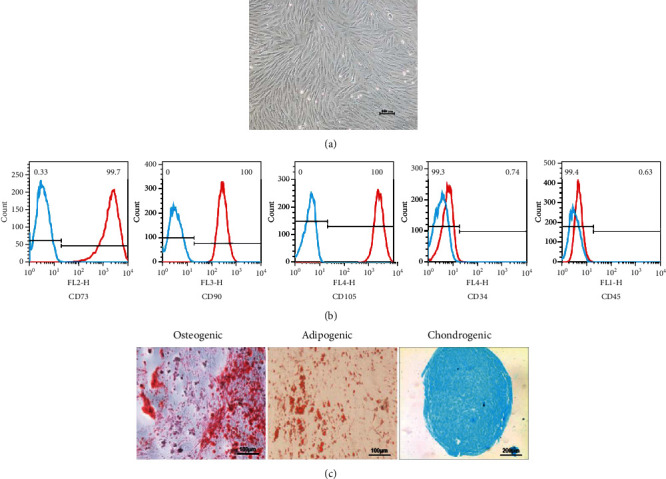
Isolation and identification of IPFP-MSC. (a) The P3 generation IPFP-MSC. (b) The results of flow cytometry. Blue line: negative control; red line: IPFP-MSCs. (c) Three-line differentiation experiments.

Primary IPFP-MSCs were extracted using the method described above and evaluated by microscopy each day thereafter. After 24 hours, we observed a small amount of adherent cellular growth. After 2 weeks, the cells had reached 80% confluency. The cells were then passaged at a ratio of 1 : 3; this allowed the MSCs to proliferate rapidly after subculture, showing a fusiform fibroblast-like appearance (Figure 1(a)). Since cells aged after multiple passages, we selected cells from passage 3 (P3) for experimentation. Flow cytometry results showed that 99% of cells expressed CD73, CD90, and CD105, while <1% of cells expressed CD34 and CD45 (Figure 1(b)). These results indicated that the extracted MSCs were consistent with previous publication standards [22]. Three-line differentiation experiments were then carried out and alizarin red staining was performed 4 weeks after osteogenic induction culture. Microscopic observation revealed the presence of scattered calcium nodules and calcified matrix. After 4 weeks of adipogenic induction culture, oil red O staining was performed; this showed that lipid droplets had formed and fused into a sheet. After 4 weeks of cartilage-induced culture, we observed the formation of cartilage pellets. Following alcian blue staining, we were able to visualize the cartilage matrix around the cells and a large amount of mucopolysaccharide (Figure 1(c)). Collectively, these results indicated that the extracted cells expressed surface proteins that were specific to MSCs and exhibited the potential to differentiate in multiple ways. Consequently, these cells were proved to be MSCs derived from IPFP (IPFP-MSCs).

### 3.2. Characterization of MSC-EXOs

**Figure 2:**
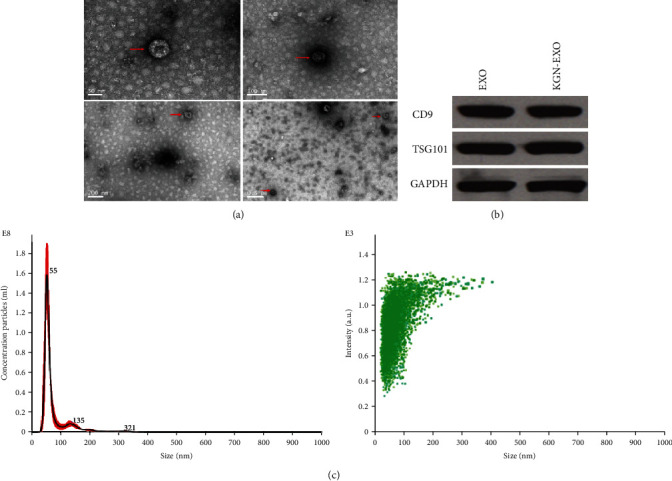
Isolation and identification of exosomes. (a) TEM results. The red arrow indicates exosomes. (b) Identification of exosome surface proteins. Western blotting showed that these exosomes were positive for the exosome-specific surface proteins CD9 and TSG101. (c) NTA test results. The size of the particles within the precipitate were predominantly 40–100 nm in diameter. Red line: range of particle concentration at different diameter sizes; black line: average concentration of particles at different diameter sizes.

Exosomes were isolated by collecting and ultracentrifuging the supernatant collected during the culture of MSCs. Transmission electron microscopy revealed that these exosomes were flat and disc shaped with a double-sided concave structure. The diameter of these cells was 40–120 nm (Figure 2(a)), thus concurring with the expected shape characteristics of exosomes. Western blotting showed that these exosomes were positive for the exosome-specific surface proteins CD9 and TSG101 (Figure 2(b)). NTA further showed that the size of the particles within the precipitate were predominantly 40–100 nm in diameter (Figure 2(c)). These results indicate that the exosomes we isolated exhibited the characteristics of exosomes and could be used for subsequent experiments.

### 3.3. Exosomes Promoted the Proliferation of Chondrocytes

**Figure 3:**
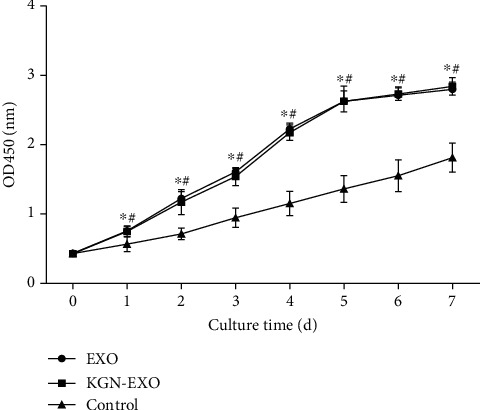
The proliferation of chondrocytes after treatment with exosomes. The proliferation of chondrocytes in the EXO group and the KGN-EXO group increased significantly compared with that in the control group. ^∗^Significant difference between the EXO group and the control group (*P* < 0.05). ^#^Significant difference between the KGN-EXO group and the control group (*P* < 0.05). d: days.

CCK-8 assays showed that the proliferation of chondrocytes in the Exo group and the KGN-Exo group increased significantly compared with the control group. There were statistical differences between the KGN-EXO group and the control group (*P* < 0.01) and also between the EXO group and the control group. There was no statistical difference between the KGN-EXO group and the EXO group (*P* > 0.05) (Figure 3). These results suggested that exosomes enhanced the proliferation of chondrocytes with or without KGN pretreatment.

### 3.4. Exosomes Induced Phenotypic Changes in Chondrocytes *In Vitro*

**Figure 4:**
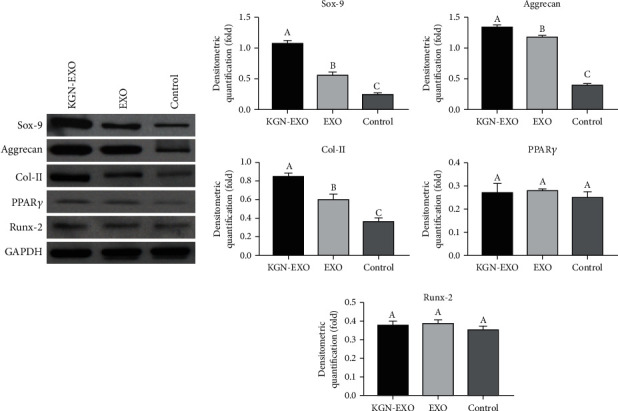
The Western blotting results showed that the expression levels of Sox-9, Aggrecan, and Col II, increased significantly after treatment with EXO and KGN-EXO when compared with those in the control group. Moreover, the KGN-EXO treatment was more effective than the EXO treatment alone (*P* < 0.05). Note that different letters above bars indicate significant differences (*P* < 0.05) while matched letter means no significant difference in each comparison among the KGN-EXO/EXO/control groups.

**Figure 5:**
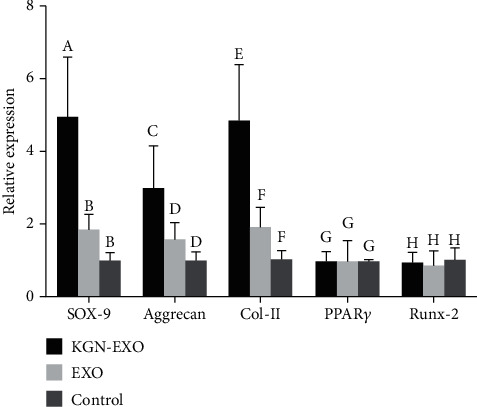
qRT-PCR results showed that the expression levels of Sox-9, Aggrecan, and Col II were significantly increased after treatment with KGN-EXO and EXO. For all three genes, there were statistical differences between the KGN-EXO group and the control group (*P* < 0.05) and statistical differences between the KGN-EXO group and the EXO group (*P* < 0.05). Note that different letters above bars indicate significant differences (*P* < 0.05) while matched letters mean no significant difference in each comparison among the KGN-EXO/EXO/control groups.

In order to verify whether exosomes could influence the expression of intracellular proteins and genes associated with cartilage, we conducted several *in vitro* experiments. Western blotting showed that the expression levels of Sox-9, Aggrecan, and Col II increased significantly after treatment with EXO and KGN-EXO when compared with the control group. Moreover, the KGN-EXO treatment was more effective than the EXO treatment alone (*P* < 0.05, Figure 4). qRT-PCR showed that the expression levels of *Sox-9*, *Aggrecan*, and *Col II* were significantly increased after treatment with KGN-EXO and EXO (Figure 5). For all three genes, there were statistical differences between the KGN-EXO group and the control group (*P* < 0.05) and statistical differences between the KGN-EXO group and the EXO group (*P* < 0.05). Although the expression levels of the three genes were increased in the EXO group compared with the control group, there was no statistical difference between these two groups (*P* > 0.05). qRT-PCR found no significant difference in the gene expression of *PPARγ* and *Runx-2* when compared with that across the three groups. Western blotting also showed that the expression levels of PPAR*γ* and Runx-2 proteins were similar across the three groups. Collectively, these results showed that KGN-EXO and EXO did not improve the expression of genes or proteins related to osteogenesis or adipogenesis.

### 3.5. Macroscopic and Histological Evaluation

**Figure 6:**
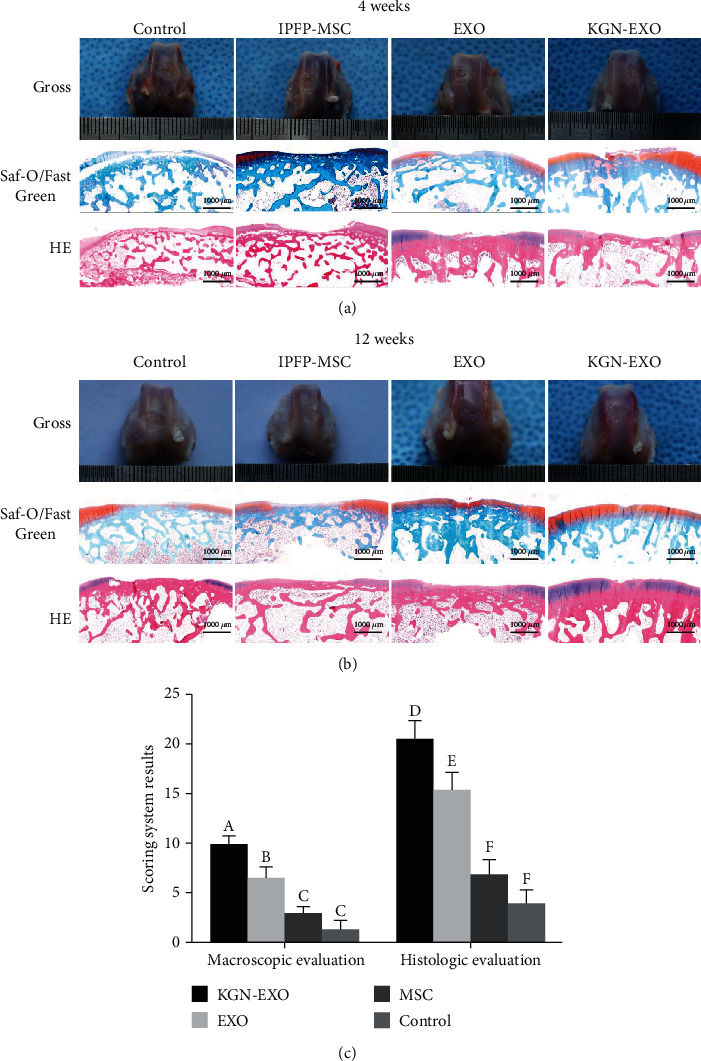
In vivo cartilage repair at 4 and 12 weeks after surgery. (a) The gross appearance, Saf-O/Fast Green and HE staining at 4 weeks. (b) The gross appearance, Saf-O/Fast Green and HE staining at 12 weeks. (c) The ICRS and histological scores at 12 weeks.

Four weeks after surgery, there was almost no repair tissue in either the control group or the IPFP-MSC group; the boundary with the surrounding normal cartilage tissue was obvious. The defect area was extremely uneven and no new cartilage had been formed. A small number of cartilage-like structures had formed in the defect area of the EXO group; this formed a connection with the surrounding normal articular cartilage tissue and gathered towards the center. More cartilage tissue had formed in the KGN-EXO group; the surface was relatively flat and was well connected with the surrounding normal articular cartilage thus showing good levels of repair (Figure 6(a)). H&E staining indicated that almost no cartilage-like structures had formed in the control group and the IPFP-MSC group. Only a small amount of cartilage-like tissue had formed at the bottom of the defect site in the EXO group; the thickness of the regenerated tissue was less than 50% than that of the normal cartilage in the surrounding area. Hyaline cartilage had formed in the KGN-EXO group; the thickness of the regenerated tissue was significantly greater than that of the other three groups. S&F staining showed that only the regenerated tissues in the KGN-EXO group showed strong, positive, and uniform Safranin O staining, thus suggesting that the proteoglycan content in the regenerated tissue of the KGN-EXO group was similar to that of normal cartilage (Figure 6(a)).

Twelve weeks after surgery, the control group showed almost no regeneration of cartilage tissue and the defect area was clearly evident. The surface was uneven with poor levels of integration with the surrounding normal articular cartilage tissue. The defect area in the IPFP-MSC group was predominantly filled with fibrous connective tissue; a small amount of cartilage tissue had formed at the edge of the defect, and the surface was uneven. The repaired tissue was partially connected with the surrounding normal articular cartilage tissue. In the EXO groups, the defect area was filled by a large area of regenerated cartilage-like tissue. The defect site had been filled with small cracks evident in the repaired tissue. The surface of this regenerated tissue was relatively flat and the repaired tissue had integrated with the surrounding normal articular cartilage, although the boundary was still obvious. The surface of the defect site in the KGN-EXO group was smooth and flat and was almost covered by regenerated cartilage tissue. Very few cracks were evident in the repaired tissue, which showed good integration with the surrounding normal cartilage; it was difficult to determine the boundary (Figure 6(b)). The ICRS scores for the KGN-EXO group (9.94 ± 0.87) were significantly higher than those for the other three groups (*P* < 0.01; Figure 6(c)). The ICRS scores for the EXO group (6.56 ± 1.10) were significantly higher than those for the control group (1.33 ± 0.84) and the IPFP-MSC group (3.00 ± 0.69) (*P* < 0.01). The ICRS scores for the IPFP-MSC group were higher than those for the control group, although the difference was not statistically significant (*P* > 0.05). These results suggested that the KGN-EXO treatment had the strongest ability to repair cartilage defects *in vivo* and was significantly better than any of the other three groups. H&E staining showed that a small amount of nonchondroid tissue had formed in the control group. There were only very minimal amounts of hyaline cartilage structure in the repaired tissue and the Safranin O staining was not significantly positive. A large number of cracks were observed in the regenerated tissue which showed poor integration with the surrounding normal cartilage. In the IPFP-MSC group, we observed moderate tissue regeneration; the regeneration has occurred unevenly and the hyaline cartilage structure was thinner than that in the KGN-EXO group; many cracks were evident in this tissue. Safranin O staining was little and not fully integrated with the surrounding normal cartilage. More explant tissue regeneration was evident in the EXO group; more than 70% of the defect area had been filled. Hyaline cartilage tissue was visible inside the defect area and there were few cracks. Safranin O staining was very prominent, indicating that this tissue contained a significant proportion of proteoglycans. The regenerated tissue was well integrated with the surrounding normal cartilage, and a clear boundary was evident. In the KGN-EXO group, the defect site had been almost completely repaired; the surface was flat and smooth. We also observed a notable proportion of hyaline cartilage-like tissue with no obvious cracks. Safranin O staining showed uniform and strong positive staining, suggesting that this tissue contained a large proportion of proteoglycans. Furthermore, the repaired tissue was fully integrated with surrounding normal cartilage and it was difficult to visualize the boundaries. Histological scoring (Figure 6(c)) at 12 weeks after surgery showed that the histological scores of the KGN-EXO group (20.56 ± 1.91) were significantly higher than those of the other 3 groups (*P* < 0.01). The histological scores of the EXO group (15.44 ± 1.79) were also significantly higher than those of the control group (3.94 ± 1.43) and the IPFP-MSC group (6.89 ± 1.49) (*P* < 0.01). The score of the IPFP-MSC group was higher than that of the control group, but this was not statistically significant (*P* > 0.05). Collectively, these results showed that the exosomes derived from IPFP-MSCs that had been pretreated with KGN possessed a strong ability to promote the repair of cartilage defects *in vivo.*

## 4. Discussion

In this study, we used KGN to pretreat IPFP-MSCs and then successfully isolated exosomes from the supernatant of MSCs by ultracentrifugation. We then evaluated the characteristics of the isolated exosomes by transmission electron microscopy, surface protein identification, and NTA assays. *In vitro* experiments demonstrated that the exosomes derived from MSCs could significantly enhance the proliferation of chondrocytes, but KGN pretreating method could not increase the capacity of MSC exosomes to promote proliferation additionally. The MSC exosomes pretreated with KGN could significantly promote the expression of cartilage-associated proteins and genes compared with non-KGN-pretreated exosomes. In the *in vivo* experiments, better cartilage repair and a large amount of hyaline cartilage-like tissue regeneration in the defect site were found in the KGN-EXO treatment group compared with the other three groups. The surface of the repaired tissue was smooth, flat, and well integrated with the surrounding normal cartilage. No obvious boundary was observed, and Safranin O staining was strong and positive. Macroscopic evaluation and histological scoring also proved that the KGN-EXO group had the best overall efficacy with regard to the repair of cartilage defects in our rabbit models.

MSCs are commonly used for the regeneration of lesions in articular cartilage. Indeed, results derived from both basic science and clinical research have reported promising results using this technique [23]. The most commonly used MSCs in earlier researches were BM-MSCs (bone marrow mesenchymal stem cells), AD-MSCs (adipose-derived stem cells), SMSCs (synovial mesenchymal stem cells), and UC-MSCs (umbilical cord mesenchymal stem cells). The infrapatellar fat pad is a type of fat that is situated under and behind the patella bone within the knee and has traditionally been considered as a cushion to buffer forces in the joint. However, an increasing body of evidence now supports the fact that mesenchymal stem cells can be extracted from the infrapatellar fat pad and exhibit far better chondrogenic ability than other forms of MSCs [24]. Koh and Choi were the first to use infrapatellar fat pad-derived mesenchymal stem cell therapy to treat knee osteoarthritis; over the short-term, the results from this study were encouraging and demonstrated that the injection of IPFP-MSCs was safe and could reduce pain and improve knee function [25]. In another study, Dragoo and Chang used arthroscopic techniques to harvest the IPFP and successfully isolate adipose-derived MSCs, thus making it easier for the application of IPFP-MSCs in clinic. Neri et al. subsequently used *in vitro* experiments to demonstrate that IPFP-MSCs derived from patients with OA still met the criteria to be considered as MSCs and were suitable and safe for the regeneration of cartilage [26]. Initially, it was thought that cell replacement therapy would be the best protocol to apply MSCs to repair cartilage lesions. This concept was based on the ability of these cells to undergo chondrogenic differentiation and to secrete PGs and collagen II, which are the essential components of tissue [12, 27]. It also indicates that MSCs possess immunomodulatory properties that may help to reduce the loss of cartilage [28]. However, recent studies showed that the principal source of repair tissue is derived from endogenous cells following the intra-articular transplantation of MSCs, thus implying that paracrine effects may be predominantly responsible for the manner in which MSCs induce cartilage regeneration [27, 29].

The paracrine effects of MSCs predominantly include soluble factors and extracellular vesicles. In particular, exosomes released by MSCs have been shown to influence cartilage regeneration [13, 30, 31]. Given that there is still many limitations relating to the use of MSCs in clinics to treat cartilage lesions, such as ethical issues and policy limitations, it is quite necessary and important that we continue to seek a cell-free treatment to promote cartilage repair, which could be easier to translate for clinical application [11]. Therefore, exosomes represent a promising MSC-based cell-free method to induce cartilage regeneration. In a previous study, Wang et al. reported that secretory factors from UC-MSCs could regulate the differentiation of MSCs [32]. In a subsequent paper, Huang et al. proposed that exosomes derived from MSCs could represent an alternative treatment for cartilage repair in the form of a cell-based tissue engineering strategy [33]. Zhang et al. subsequently demonstrated that exosomes from ESCs could promote osteochondral regeneration [30] while Cosenza et al. showed that exosomes from BM-MSCs could protect the cartilage and bone from degradation in OA [34]. A subsequent study by Tao et al. found that exosomes from SMSCs had significant potential to prevent the progression of OA and that the efficacy of this technique could be significantly enhanced by the overexpression of miR-140-5p in SMSCs [35]. In another paper, Wu et al. suggested that miR-100-5p-abundant IPFP-MSC-EXOs could protect articular cartilage by inhibiting mTOR in OA [36]. Qi et al. proved that exosomes from BMSCs could inhibit mitochondrial dysfunction-induced apoptosis in chondrocytes via p38, ERK, and Akt pathways [37]. Thus, many studies have demonstrated the advantages of MSC-EXOs for cartilage repair and the prevention of OA. However, the components and function of exosomes are quite susceptible for variability and can differ when extracted from different cell types or the same cell types under different conditions [13, 29]. This inconsistency makes it difficult to consider the results arising from previous studies, but also gives us a chance to pretreat the original cells to enhance the subsequent function of the exosomes. For example, Kato et al. used IL-1 to stimulate synovial fibroblasts and found that exosomes isolated from IL-1-stimulated synovial fibroblasts could induce more osteoarthritic changes in articular chondrocytes than those without IL-1 stimulation, thus proving that the function of exosomes can be regulated by pretreatment [38]. KGN is a small heterocyclic compound that exhibits a strong ability to induce MSCs to differentiate into chondrocytes [18, 39]. In the present study, we used KGN-pretreated IPFP-MSCs to investigate whether this action influenced the ability of the exosomes to induce the differentiation of chondrocytes. We found that exosomes from IPFP-MSCs could significantly promote the proliferation of chondrocytes, thus concurring with previous reports [40]. No significant change was observed when IPFP-MSCs were pretreated with KGN or not. We also found that exosomes derived from IPFP-MSCs could enhance the anabolic effects of chondrocytes and reduce catabolic effects by increasing the expression of SOX-9, Aggrecan, and COL-2 and by reducing the expression of MMPs, which also concurred with previous studies [34, 40, 41]. Furthermore, exosomes derived from KGN-pretreated IPFP-MSCs could significantly enhance anabolic effects and reduce catabolic effects when compared with exosomes derived from IPFP-MSCs without pretreatment. This implies that exosomes derived from KGN-pretreated MSCs may be more beneficial for cartilage repair. We also performed *in vivo* experiments to further verify our hypothesis.

There are some limitations associated with this study that need to be considered. We observed that KGN pretreatment could enhance the function of MSC-EXO for cartilage regeneration, but we were not able to elucidate the specific mechanisms responsible for this effect. Future studies would be needed to identify such mechanisms. The results of previous studies imply that microRNA changes may be the most likely mechanism [35, 36, 42]. Furthermore, we directly injected MSC-EXO without a carrier. Future studies will need to identify a reliable carrier for EXO in order to promote their sustained release, although exosome could be a promising cell-free therapy used in clinical practice without many policy limitations like cell therapy. However, exosome-related research still mainly stay in the laboratory. It should not be used in clinical practice until the safety and efficacy of exosome is verified in clinical trials. Also, it should not be ignored that the cost of exosome therapy is even more than the cost of cell therapy, which could be the potential limitation to translate the exosome method into clinical practice.

## 5. Conclusion

Our study demonstrated, for the first time, that exosomes isolated from infrapatellar fat pad mesenchymal stem cells can be pretreated with KGN to induce stronger chondrogenic capability. These exosomes effectively promoted the proliferation and expression of chondrogenic proteins and genes in chondrocytes. These exosomes were also able to promote the repair of articular cartilage defects in a very effective manner. We propose that these exosomes can be used as a potential therapeutic method in the future.

## Figures and Tables

**Table 1:** International Cartilage Repair Society macroscopic evaluation of cartilage repair.

Categories	Score
Degree of defect repair	
In level with surrounding cartilage	4
75% repair of defect depth	3
50% repair of defect depth	2
25% repair of defect depth	1
No repair of defect depth	0
Integration to border zone	
Complete integration with surrounding cartilage	4
Demarcation border < 1 mm	3
Three-quarters of graft integrated, one-quarter with a notable border > 1 mm in width	2
One-half of graft integrated with surrounding cartilage, one-half with a notable border > 1 mm	1
From no contact to one-quarter of graft integrated with surrounding cartilage	0
Macroscopic appearance	
Intact smooth surface	4
Fibrillated surface	3
Small, scattered fissures or cracks	2
Several small or few large fissures	1
Total degeneration of grafted area	0
Overall repair assessment	
Grade I: normal	12
Grade II: nearly normal	8–11
Grade III: abnormal	4–7
Grade IV: severely abnormal	0–3

**Table 2:** The modified O'Driscoll histologic score.

Characteristic		Score
% hyaline cartilage	80–100	8
	60–80	6
	40-60	4
	20-40	2
	0-20	0
Structural characteristics		
Surface irregularity	Smooth and intact	2
	Fissures	1
	Severe disruption, fibrillation	0
Structural integrity	Normal	2
	Slight disruption, including cysts	1
	Severe lack of integration	0
Thickness	100% of normal adjacent cartilage	2
	50% to 100% or thicker than normal	1
	0–50%	0
Bonding to adjacent cartilage	Bonded at both ends of graft	2
	Bonded at one end/partially both ends	1
	Not bonded	0
Freedom from cellular changes of degeneration	Normal cellularity, no clusters	2
	Slight hypocellularity, <25% chondrocyte clusters	1
	Moderate hypocellularity, >25% clusters	0
Freedom from degenerate changes in adjacent cartilage	Normal cellularity, no clusters, normal staining	3
	Normal cellularity, mild clusters, moderate staining	2
	Mild or moderate hypocellularity, slight staining	1
	Severe hypocellularity, slight staining	0
Reconstitution of subchondral bone	Complete reconstitution	2
	Greater than 50% reconstruction	1
	50% or less reconstruction	0
Bonding of repair cartilage to de novo subchondral bone	Complete and uninterrupted	2
	<100% but >50% reconstruction	1
	<50% complete	0
Safranin O staining	>80% homogeneous positive stain	2
	40%–80% homogeneous positive stain	1
	<40% homogeneous positive stain	0
	Total score	Max 27

## Data Availability

All data is available from the corresponding author YZ (drzhouyiqin@163.com) when requested.
